# Supplementary material for: Molecular mechanisms of Holliday junction branch migration catalyzed by an asymmetric RuvB hexamer
Source: Nat Commun. 2023 Jun 15;14:3549. doi: 10.1038/s41467-023-39250-6 (PMC10272136; doi:10.1038/s41467-023-39250-6)
Supplement: Supplementary file 1 — Supplementary Information [file 41467_2023_39250_MOESM1_ESM.pdf]

Molecular Mechanisms of Holliday Junction Branch Migration Catalyzed by an Asymmetric RuvB Hexamer

Anthony D. Rish[1,2,3,6], Zhangfei Shen[2,3,6], Zhenhang Chen[2,3,5], Nan Zhang[3,4], Qingfei Zheng[1,2,3,4], Tian-Min Fu[1,2,3,*]

This PDF file includes the following:

Supplementary Figures 1-10

Supplementary Table 1

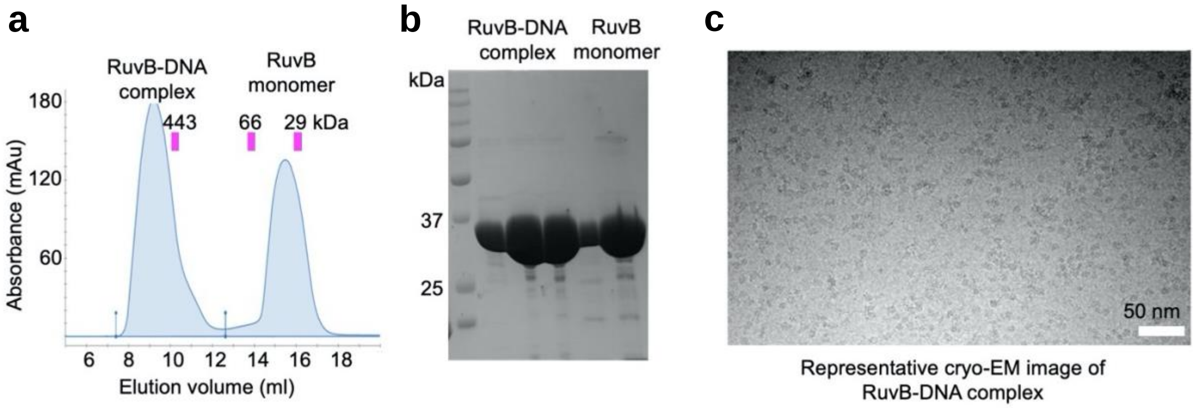

Supplementary Figure 1. Purification and Imaging of the RuvB-DNA complex. a Gel filtration profile of the RuvB-DNA complex on a Superdex S200 10/300 GL Increased column. **b** A representative SDS-PAGE gel showing the purified RuvB-DNA complex that had been replicated at least three times. **c** A representative cryo-EM image of the RuvB-DNA complex from a stack of 6,870 micrographs.

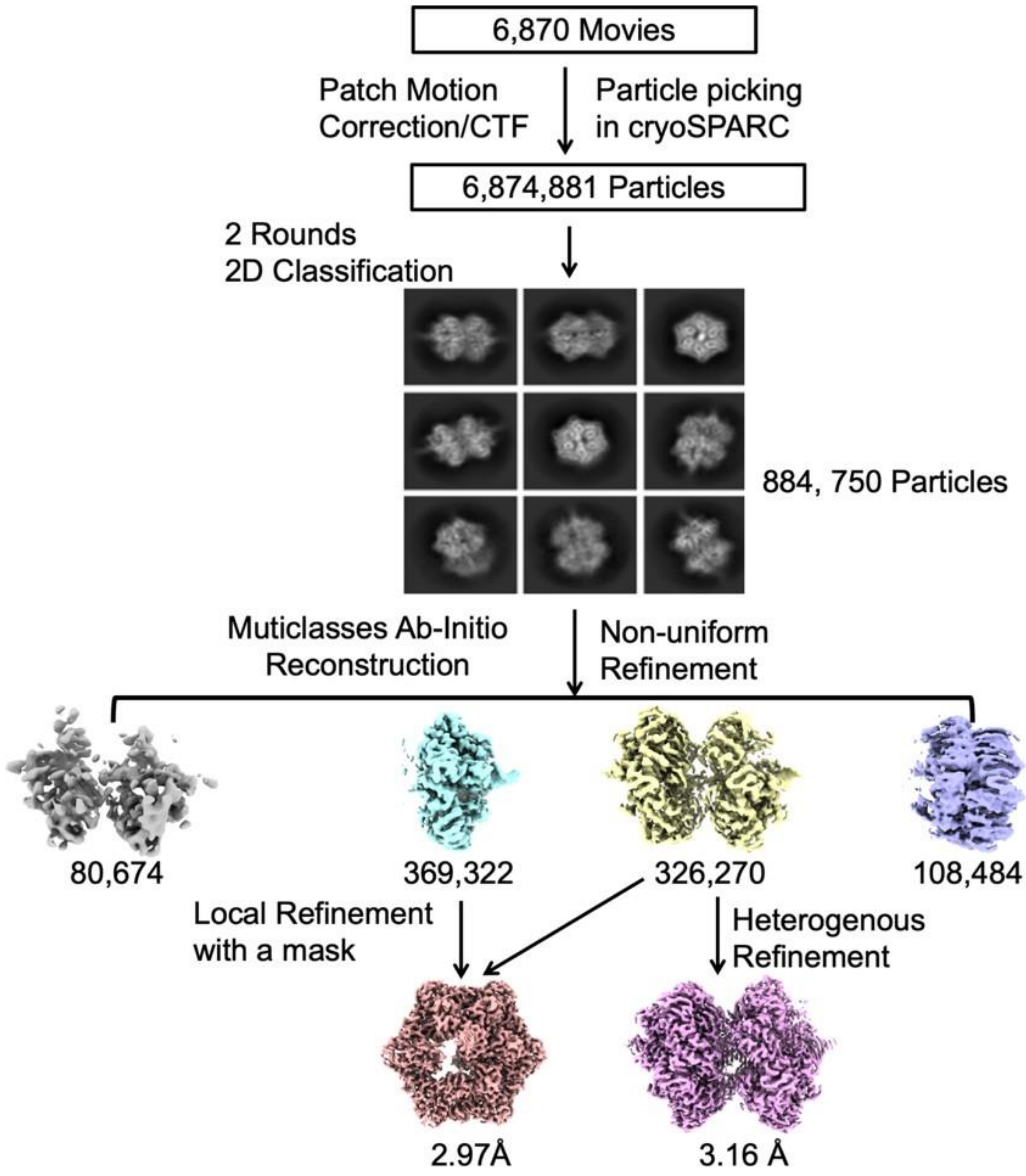

Supplementary Figure 2. Workflow of the RuvB-DNA complex 3D reconstruction using cryoSPARC.

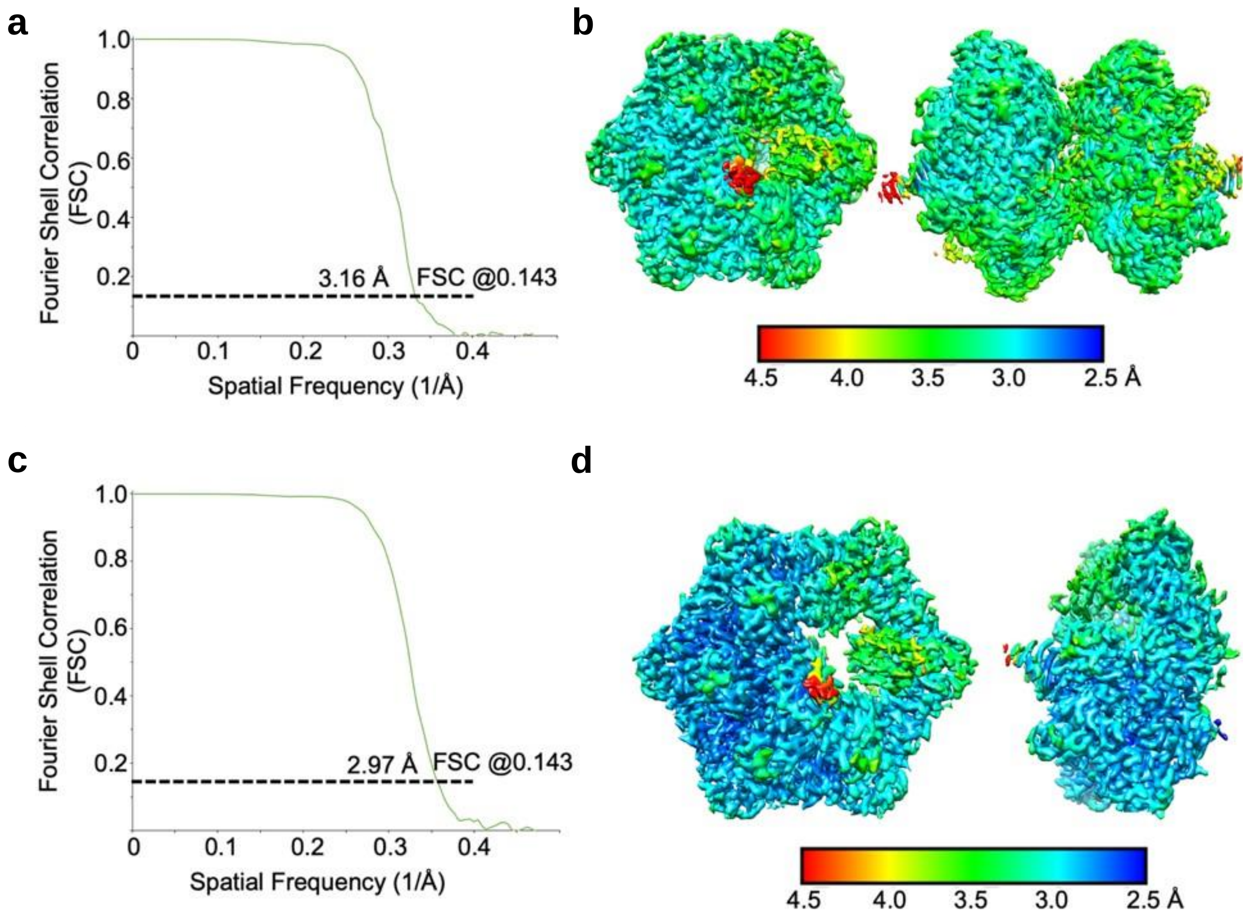

Supplementary Figure 3. Resolutions of the RuvB-DNA complexes. a Fourier shell correlation (FSC) curve of 3D reconstructed RuvB dodecamer. **b** Local resolutions of the RuvB dodecamer. Resolutions are color-coded by scale bars. **c** Fourier shell correlation (FSC) curve of 3D reconstructed RuvB hexamer. **d** Local resolutions of the RuvB hexamer. Resolutions are color-coded by scale bars.

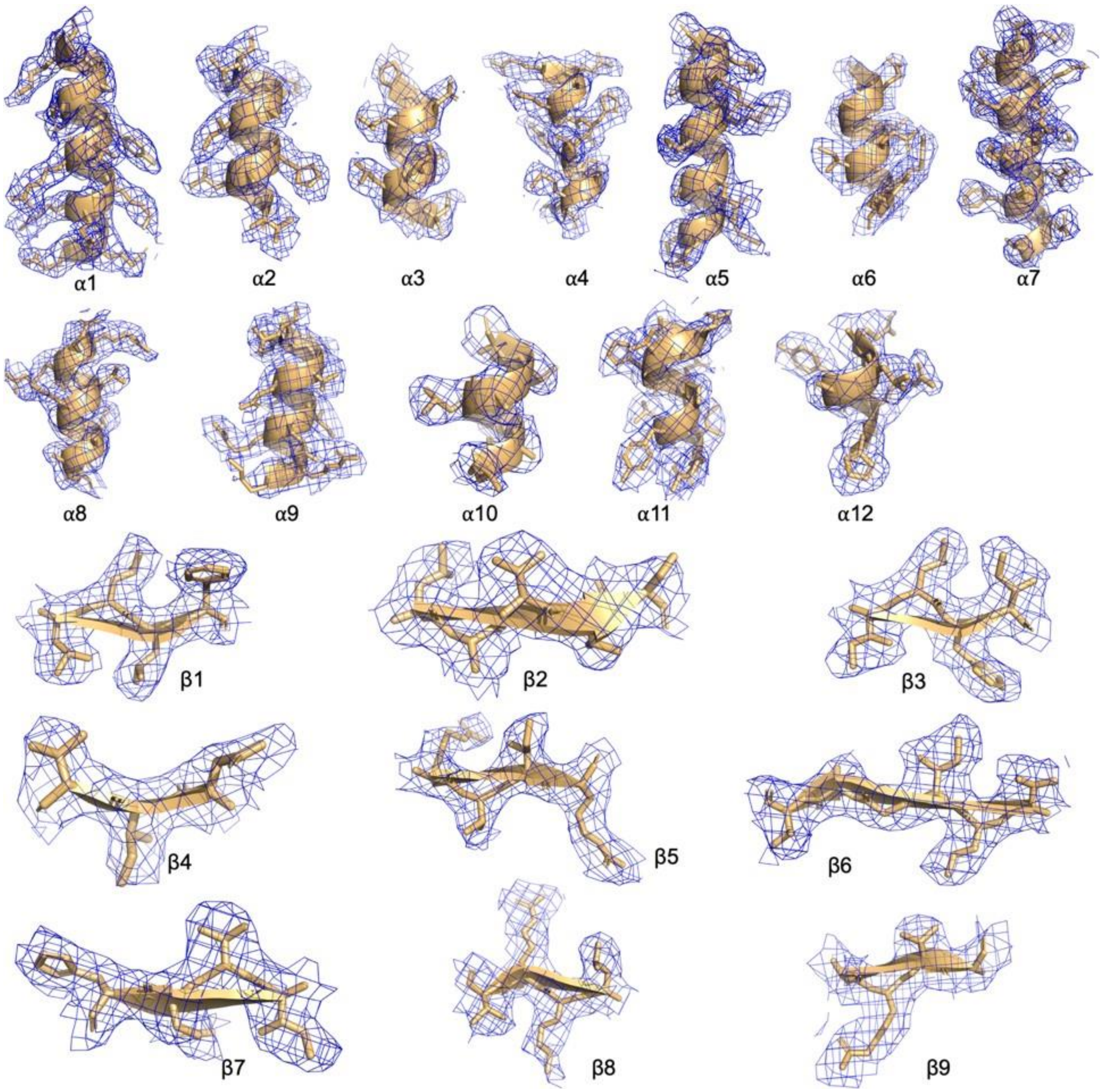

Supplementary Figure 4. Cryo-EM Density of RuvB. Representative segments of RuvB cryo-EM density map (blue mesh) with the final atomic model (wheat) (2.0 $\boldsymbol{\sigma}$).

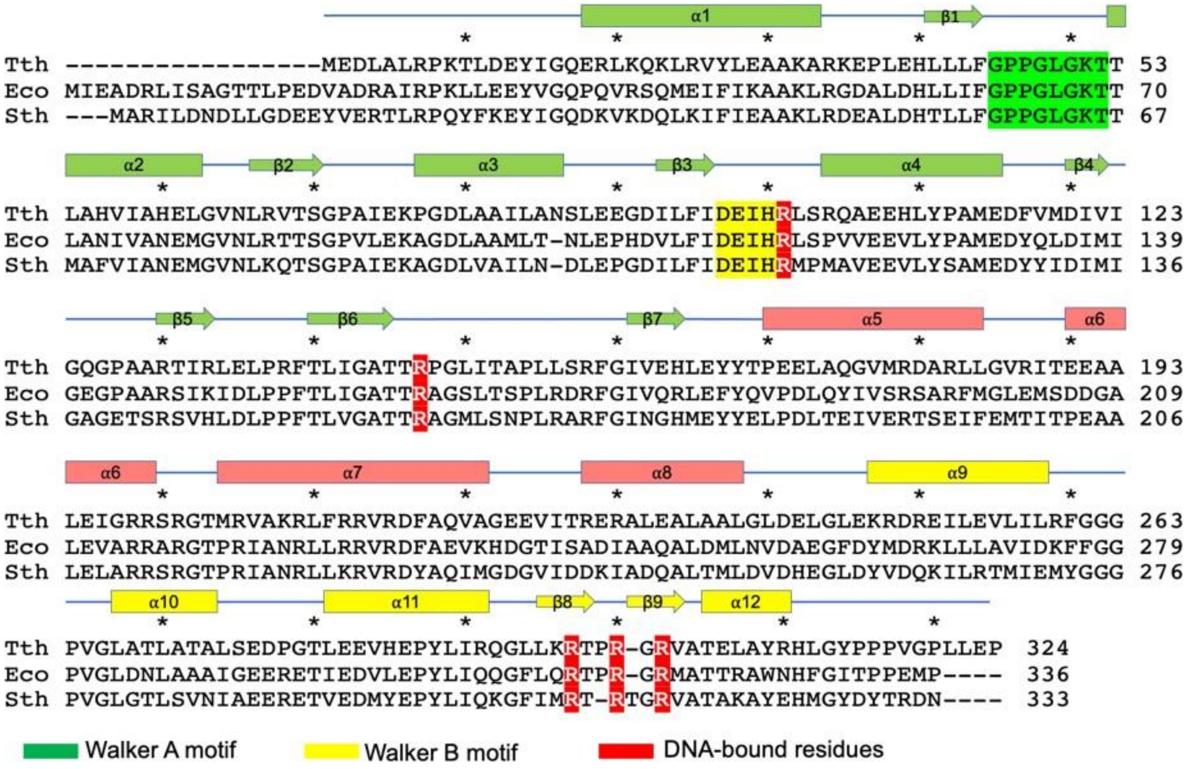

Supplementary Figure 5. Sequence Alignment of RuvB from Different Species. Sequence alignment of RuvB from *Thermus thermophilus* HB8 (Tth), *Escherichia coli* K12 (Eco*)*, and *Streptococcus thermophilus* (Sth) with secondary structural elements labeled above. NTD was highlighted in green, MD in salmon, and CTD in yellow. Walker A motif, Walker B motif, and arginine residues critical for coordinating DNA were highlighted in green, yellow, and red, respectively.

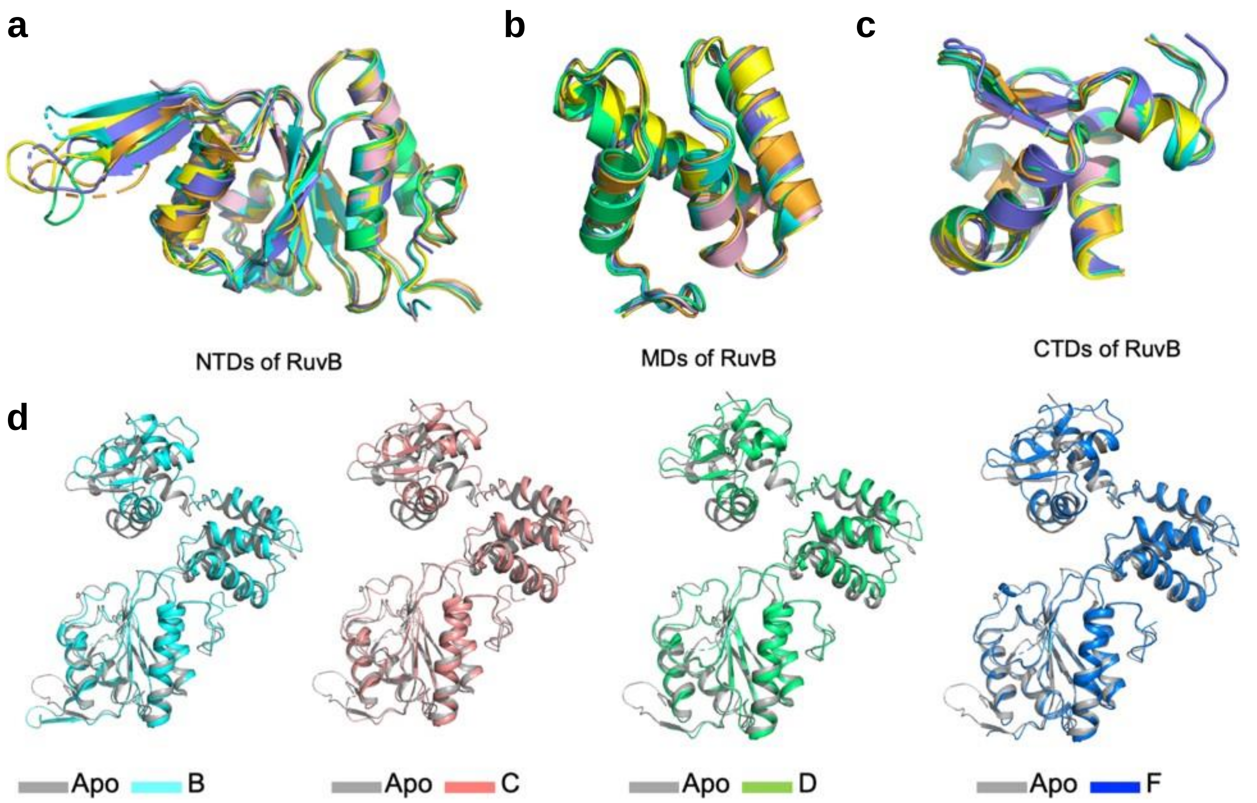

Supplementary Figure 6. Structural Domain Comparison. a Overlaid structures of RuvB N-terminal domains from the six RuvB protomers. **b** Overlaid structures of RuvB middle domains from the six RuvB protomers. **c** Overlaid structures of RuvB C-terminal domains from the six RuvB protomers. **d** Structural comparisons of RuvB protomers B (cyan), C (salmon), D (green), F (blue) with the crystal structure of RuvB (1HQC) (grey).

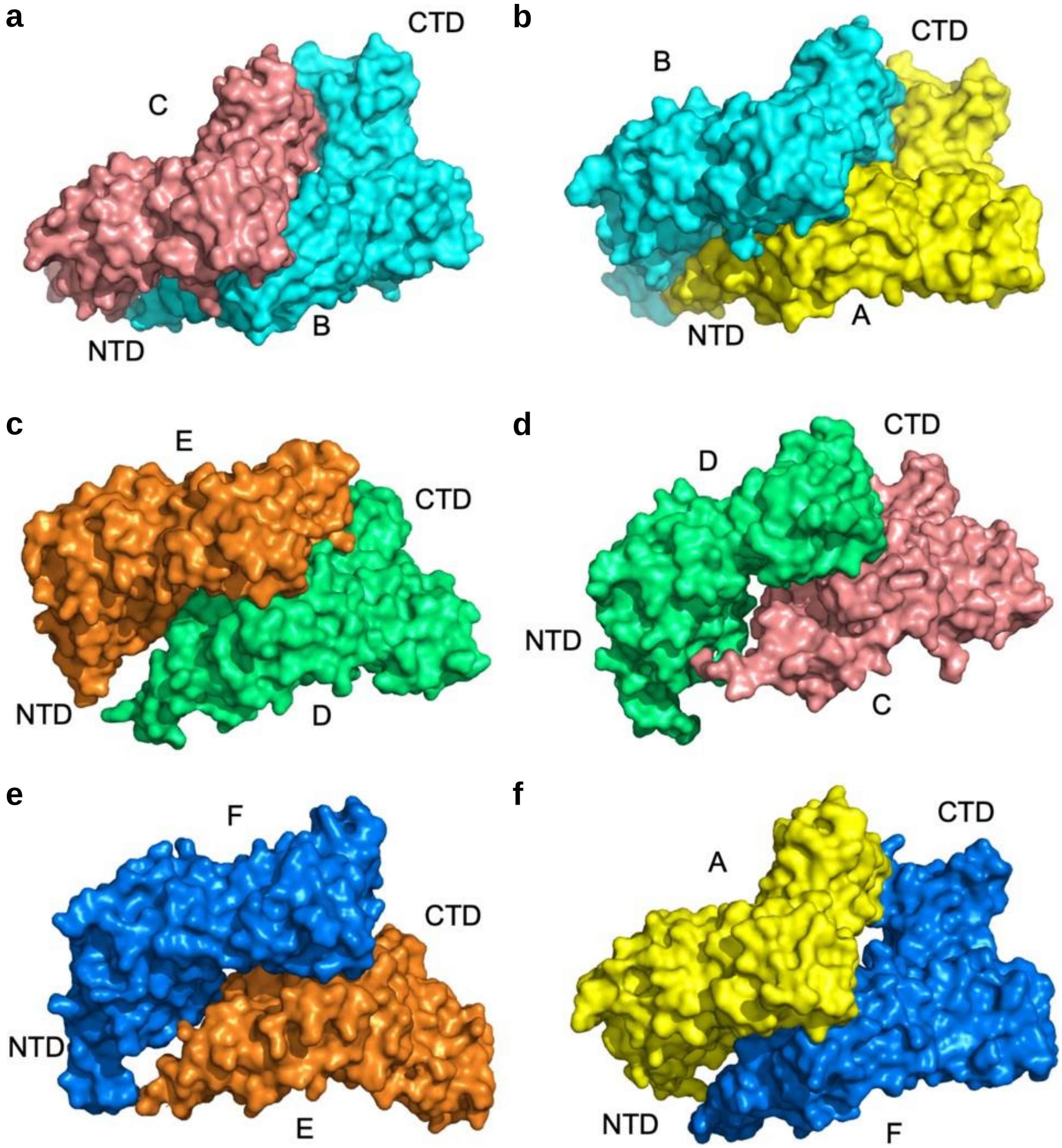

Supplementary Figure 7. Surface Representation of RuvB Dimers. Surface representation of RuvB dimers **a** BC, **b** AB, **c** DE, **d** CD, **e** EF, and **f** FA, with protomers A, B, C, D, E, F colored in yellow, cyan, salmon, green, orange, and blue, respectively

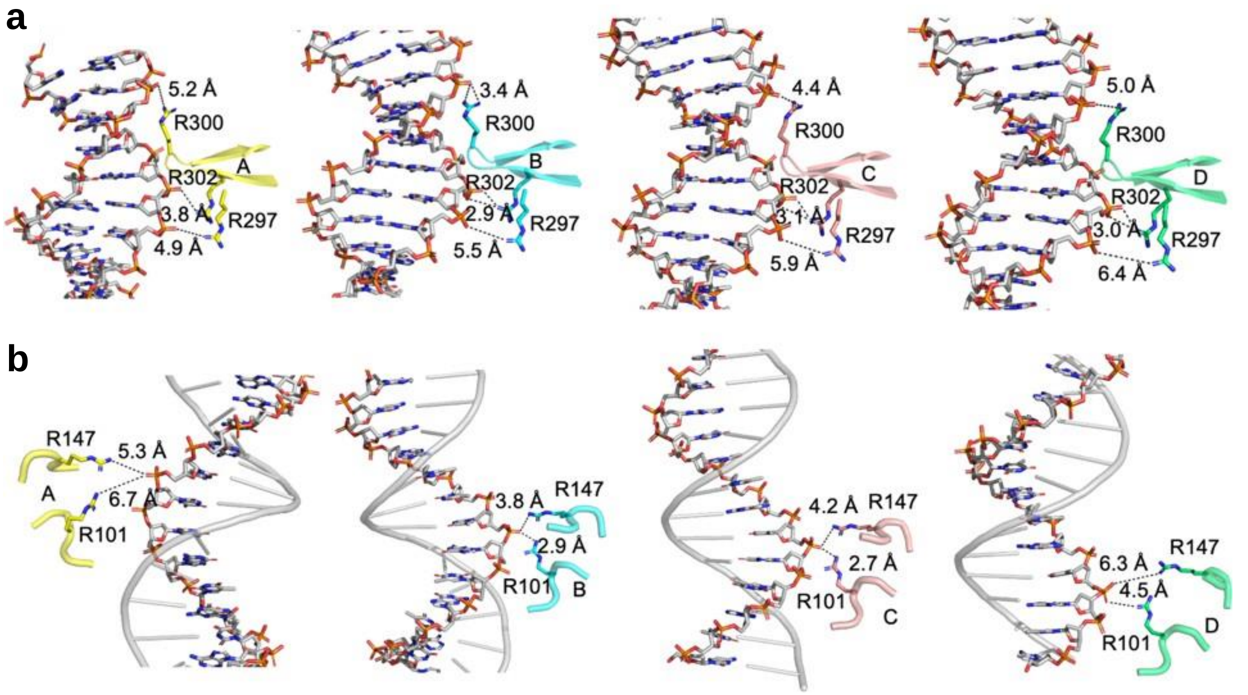

Supplementary Figure 8. Detained Interactions between RuvB and dsDNA. a Ribbon diagram showing detailed interactions between Arg297, Arg300, Arg302, and DNA backbone with distances between arginine residues and phosphate groups labeled. **b** Ribbon diagram showing detailed interactions between Arg101, Arg147, and DNA backbone with distances between arginine residues and phosphate groups labeled. Chains A, B, C, and D are colored yellow, cyan, salmon, and green respectively. DNA is shown as grey sticks or colored heteroatoms atoms.

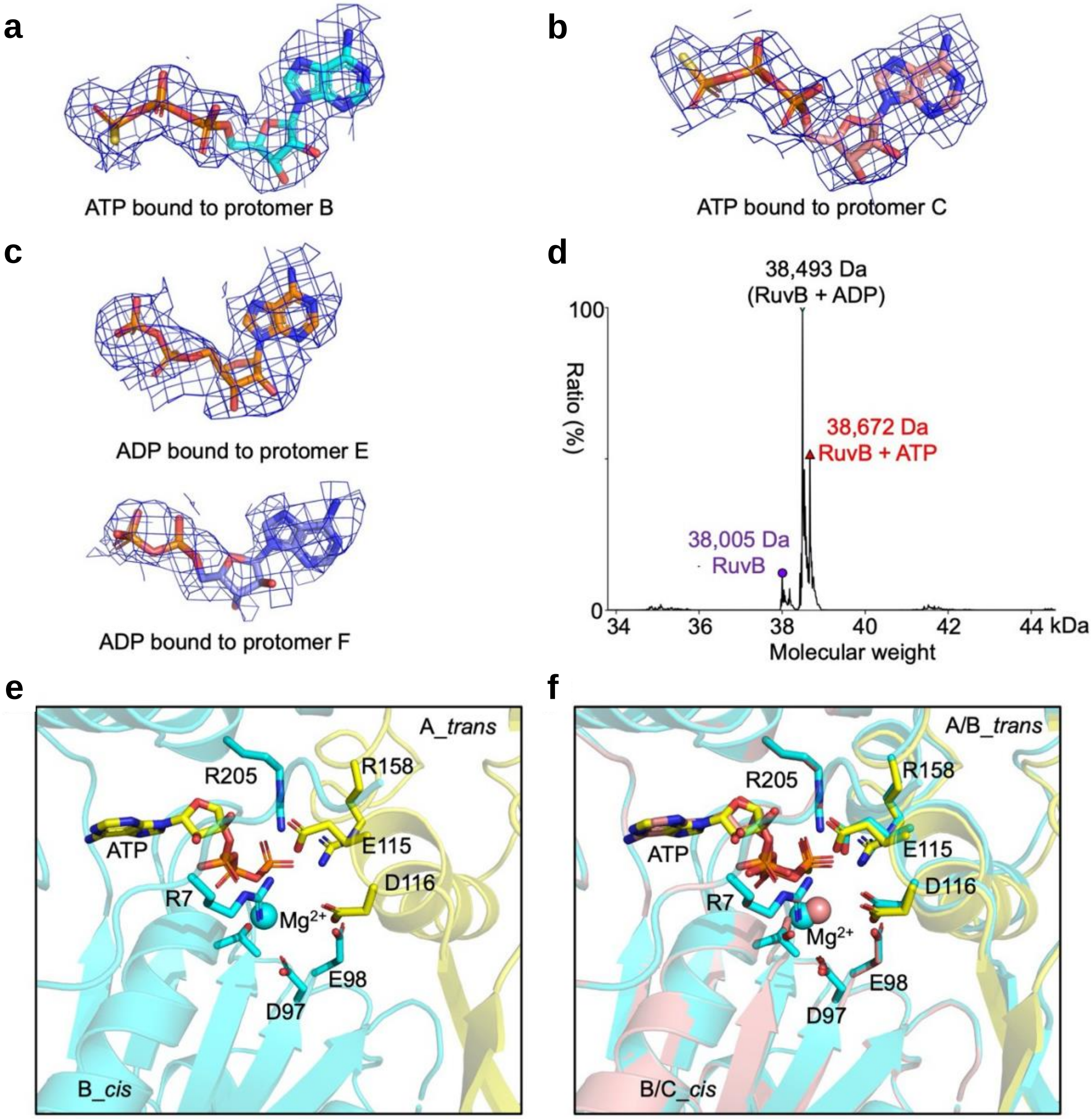

Supplementary Figure 9. Nucleotides and ATP Catalytic Centers in RuvB. a ATP fitted to the cryo-EM density maps in protomer B (cyan) at 2.0 **σ**. **b** ATP fitted to the cryo-EM density maps in protomer C (salmon) at 2.0 **σ**. **c** ADP fitted to the cryo-EM density maps in protomers E (orange) and F (purple) at 2.0 **σ**. **d** Native mass spectrometry analysis of purified RuvB, revealing bound ADP. **e** The ATP catalytic center in the BA dimer with key residues for catalysis highlighted as sticks. Protomers B and A are shown in cyan and yellow, respectively. **f** Comparison of the ATP catalytic centers in the BA dimer and the CB dimer, revealing similar conformations. Mg^{2+} ions are shown as spheres. Protomers B, A, and C are shown in cyan, yellow, and salmon, respectively.

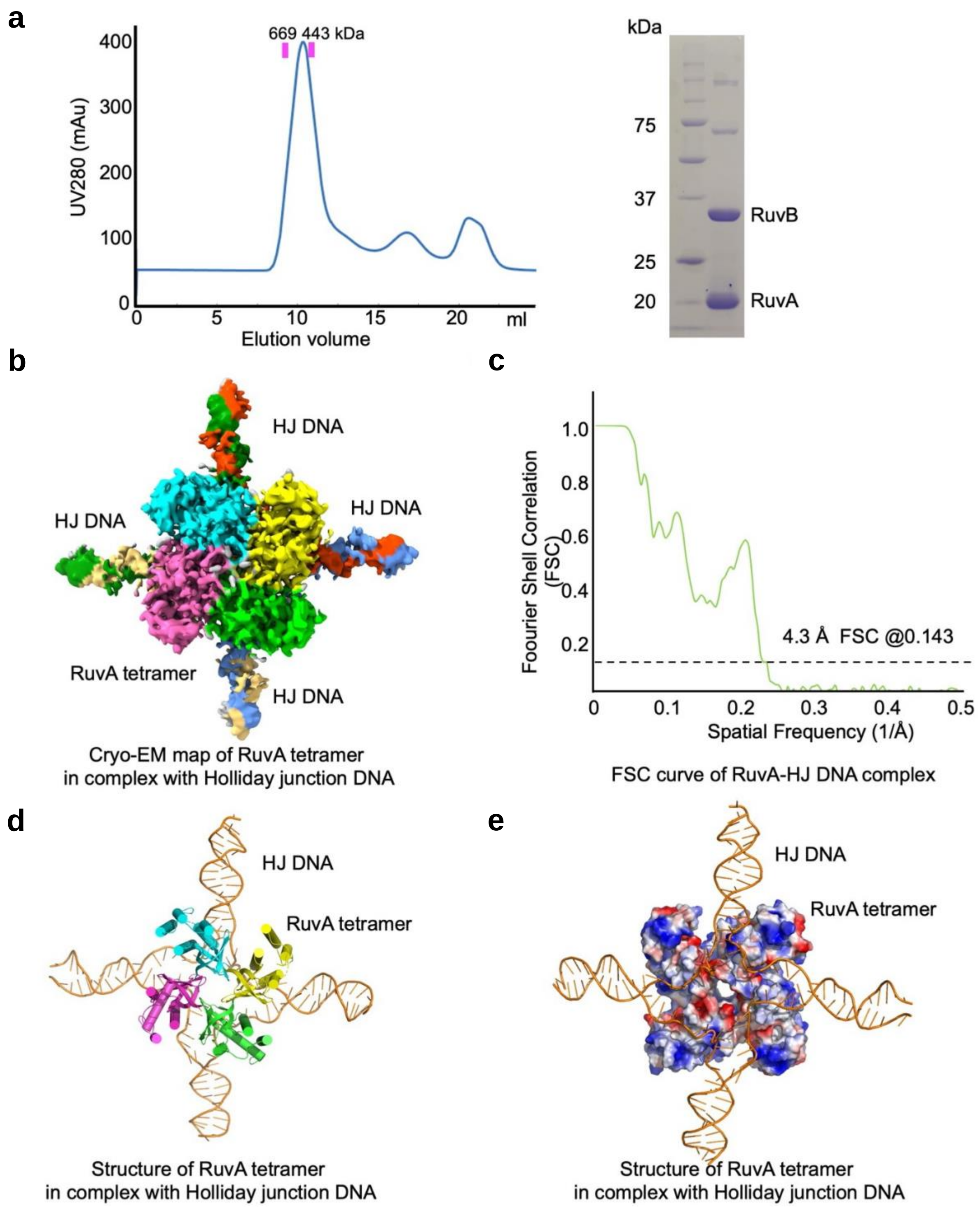

Supplementary Figure 10. Structure of RuvA-HJ DNA. a Gel filtration profile and SDS-PAGE of reconstituted RuvA-RuvB-HJ complex, that were successfully replicated at least 3 times. **b** Cryo-EM map of RuvA-HJ complex with each protomer of RuvA colored individually. **c** Fourier shell correlation (FSC) curve of 3D reconstructed RuvA-HJ complex. **d** Ribbon diagram of RuvA tetramer in complex with HJ DNA with individual subunits colored separately. **e** Electrostatic surface representation of RuvA tetramer with HJ DNA, illustrating charge-charge interactions (positive in blue and negative in red) between RuvA and HJ DNA.

Supplementary Table 1. Cryo-EM data collection, refinement, and validation statistics.

**Structures**	RuvB hexamer	RuvB dodecamer	RuvA-HJ DNA
EMDB ID	28101	28107	40036
Magnification	81,000		81,000
Voltage (kV)	300		300
Electron exposure (e–/Å^2)	50.0		50.0
Defocus range (μm)	-0.5 to -2.5		-1.5 to -2.5
Pixel size (Å)	1.08		0.95
Symmetry imposed	C1	C2	C4
Initial particles (no.)	6,874,881		33,83,678
Final particles (no.)	695,592	326,270	130,426
Map resolution (Å)<br>FSC threshold	2.97<br>0.143	3.16<br>0.143	4.34<br>0.143
**Models**			
PDB ID	8EFV	8EFY	8GH8
Initial model used (PDB)	1HQC	1HQC	7PBU & 2HOI
Map sharpening *B* factor (Å^2)	-127.6	-138.2	-203.9
Model composition<br>Non-hydrogen atoms<br>Protein residues<br>Ligands	 15295<br>1944<br>6	 31274<br>3888<br>12	 6991<br>559<br>0
R.m.s. deviations<br>Bond lengths (Å)<br>Bond angles (°)	 0.003<br>0.564	 0.004<br>0.587	 0.004<br>0.742
Validation<br>MolProbity score<br>Clashscore<br>Poor rotamers (%)	 1.68<br>7.07<br>0.00	 1.69<br>7.33<br>0.00	 2.12<br>9.74<br>0.00
Ramachandran plot<br>Favored (%)<br>Allowed (%)<br>Disallowed (%)	 95.81<br>4.19<br>0.00	 95.78<br>4.22<br>0.00	 87.57<br>12.25<br>0.18
