# Supplementary material for: Molecular mechanisms of Holliday junction branch migration catalyzed by an asymmetric RuvB hexamer
Source: Nat Commun. 2023 Jun 15;14:3549. doi: 10.1038/s41467-023-39250-6 (PMC10272136; doi:10.1038/s41467-023-39250-6)
Supplement: Supplementary file 3 — Description of Additional Supplementary Files [file 41467_2023_39250_MOESM3_ESM.pdf]

Description of Additional Supplementary Files

File Name: Supplementary Movie 1
Description: Mechanisms of DNA translocation by the RuvB hexamer
